# Sustainability of residential environmental interventions and health outcomes in the elderly

**DOI:** 10.1186/s40733-020-00066-6

**Published:** 2020-11-02

**Authors:** David A. Turcotte, Susan Woskie, Rebecca Gore, Emily Chaves, Kelechi L. Adejumo, Kim-Judy You

**Affiliations:** 1 Economics Department and Center for Community Research and Engagement, University of Massachusetts Lowell, Mahoney Hall Suite 212, 870 Broadway St., Lowell, MA 01854 USA; 2 Public Health Department, University Massachusetts Lowell, One University Avenue, Lowell, MA 01854 USA; 3 Biomedical Engineering, University Massachusetts Lowell, One University Avenue, Lowell, MA 01854 USA; 4 Center for Community Research and Engagement, University of Massachusetts Lowell, Mahoney Hall Suite 212, 870 Broadway St., Lowell, MA 01854 USA

**Keywords:** Healthy housing, Asthma, Environmental health, Aging in place

## Abstract

**Background:**

Research has documented that housing conditions can negatively impact the health of residents. Asthma has many known indoor environmental triggers including dust, pests, smoke and mold, as evidenced by the 25 million people in the U.S. population who have asthma. The paper describes a follow-up study involving elder adults with asthma who participated in a multifaceted home educational and environmental intervention shown to produce significant health benefits. On average the time between the end of the prior intervention study and the follow-up was 2.3 years. The objective of this study was to evaluate whether improvements in environmental conditions and health outcomes resulting from the original Older Adult Study (OAS, multifaceted educational and environmental interventions) would be maintained or decline over time for these low income seniors with asthma.

**Methods:**

Health assessment included data on respiratory health outcomes included the Saint George’s Respiratory Questionnaire (SGRQ) and Asthma Control Test from the original Older Adult Study (OAS) and this follow-up Older Adult Study (OAFS) along with health care utilization data. Environmental assessments included evaluation of asthma trigger activities (ATAs) and exposures before and after the original healthy homes intervention (questionnaire, home survey) and at this follow-up. Assessments were conducted in English, Khmer and Spanish.

**Results:**

At assessment in the Older Adult Follow-up Study (OAFS), the older adults maintained some of the health improvements gained during the OAS when compared to the OAS pre-intervention baseline. However, health outcomes declined from the OAS final assessment to the OAFS (only the SGRQ Impact scores were significantly different).

**Conclusion:**

These findings suggest that further study with a larger population is needed to determine if the significant health outcome improvements from multifaceted home educational and environmental interventions (OAS) could be more strongly maintained by providing additional follow-up “booster” interventions to this older adult population with asthma.

## Background

The World Health Organization (WHO) estimates that at least 300 million people suffer from asthma [1]. In the United States over 19.2 million adults suffer from asthma [2]. Asthma is linked to many known indoor environmental triggers including dust, pests, smoke and pets. Studies in the United States show that asthma and other reactive airway diseases are under-diagnosed among the elderly and that asthma-related morbidity and mortality among the elderly is increasing [3]. Nationally the number of older adults dying from asthma is 14 times higher compared to those 18–35 years of age [4]. Older adults 65+ are recognized as a population that is more sensitive to poor indoor air quality [5] and older persons spend up to 90% of their time indoors, often at home [4].

There is sufficient evidence that multi-trigger, multi-component interventions are effective in improving health of children with asthma [6–13]. However, there is little literature addressing the effectiveness of healthy homes interventions on elderly adults with asthma, another highly vulnerable population. The Community Preventive Services Task Force [14] reported that there is insufficient evidence for the effectiveness of healthy homes interventions with adults. The Task Force and the review by Crocker et al. [15] found only 3 intervention studies for adults with asthma and two of those were focused on asthma management education [16, 17]; neither one reported any significant changes in health or quality of life. A third study that only looked at improving housing conditions (energy efficiency upgrades) [18] found a 13% increase in quality of life scores in adults with respiratory conditions. In a randomized controlled trial with community health workers (CHWs) providing in-home self-management support of adults age 18 or above, there were fewer asthma symptoms and improved asthma control and health status in the intervention group [19]. However, the authors called for more studies to confirm the effectiveness and value of wider implementation of CHW-led in-home self-management.

In 2014–2016 we conducted the Older Adults Study (OAS) of asthma that collected baseline and follow-up data after multifaceted environmental and educational home interventions. The OAS showed statistically significant reductions in environmental asthma triggers and health improvements in the following areas: doctor visits, use of antibiotics for chest illnesses, respiratory symptoms and quality of life indicators, and asthma control (Asthma Control Test score), as well as several environmental trigger indicators [20]. This Older Adult Follow-up Study (OAFS) tests how well these improvements in environmental conditions and health outcomes were maintained over time.

## Methods

### Participants/recruitment

In the original Older Adult Study (OAS) [20], we recruited 86 eligible older adults with doctor-diagnosed asthma age 62 years or older residing in public and private subsidized housing in Lowell. For the current Older Adult Follow-up Study (OAFS), we contacted the original OAS subjects and recruited 60 participants within Lowell senior public and private housing developments with phone calls, direct mailings, and in some cases, visits to their residence. Human Subjects approval was received from the University of Massachusetts Lowell Institutional Review Board (IRB) (Approval number 17–010-TUR-XPD), which required written informed consent forms in English, Khmer, or Spanish.

In the original multifaceted environmental and educational intervention [20], individualized intervention plans were developed for each participant that included 5 visits for assessment, education and environmental interventions. Education was conducted in Khmer, Spanish and English as needed to provide information and handouts on recognizing and mitigating asthma triggers, as well as understanding asthma and its medical treatment. Environmental interventions included integrated pest management, mattress and pillow encasements for dust mites, cleaning supplies (high efficiency particulate air [HEPA] vacuum, green cleaning chemicals), and some structural interventions such as repairing ventilation, plumbing leaks, and cracks and holes that provide entry for pests.

### Asthma status measures and utilization data

All asthma status measures and utilization data instruments were administered by community health workers (CHWs) in both studies. As in the OAS we used the American version of the Saint George’s Respiratory Questionnaire (SGRQ) to evaluate respiratory symptoms and quality of life impacts from asthma and chronic obstructive pulmonary disease (COPD). This questionnaire has been validated for use with adult asthmatics and COPD patients [21–23]. It contains 50 questions in 2 parts that cover symptoms (frequency & severity), activity limitations and impacts on daily life, social functioning and psychological disturbances, representing quality of life issues. There is a scaled method of scoring that produces a summary measure (0–100) for each of the three components, with higher scores representing a higher impact, severity, or limitation. The symptom scale accounts for the frequency of coughing, sputum, shortness of breath, wheezing, and symptom-free days, as well as the frequency and duration of respiratory attacks. The activity scale accounts for the activities that cause breathlessness and are impacted by breathing problems. The impact (quality of life) scale accounts for the extent of respiratory problems, effect on work, qualities of cough and breathlessness, psychological effects of chest troubles, and psychological effects and interference of medication use. Other study tools used with our older adult participants included the 5 item Asthma Control Test (ACT) for assessing adequacy of asthma control. The test has been validated and is widely used in clinical settings [24–26]. It produces a score of 5 (poor control of asthma) to 25 (completely controlled asthma). We collected self-reported data on health care utilization in the previous year including emergency room visits, doctor visits for respiratory health, hospitalizations, and times on antibiotics for chest illness.

### Environmental checklist and questionnaire

In both studies the Environmental Assessor who accompanied the CHWs administered a self-reported environmental survey, which included questions to identify the following potential asthma triggers: pests (roaches and mice), combustion sources, moisture/mold, dust mites, furry pets, outdoor allergens, general indoor air pollution, and smoking, as well as asthma trigger behavior by occupant and visitors to the homes. We also utilized a general environmental health and safety checklist to visually observe environmental conditions within the overall home and in each room. From the environmental survey and checklist we developed several environmental asthma trigger indices including indices for cleaning, and indoor allergens. The indoor allergen index accounts for having a furry pet, wall-to-wall carpet or area rugs in the bedroom, mold anywhere in the home, feather or wool bedding for the asthmatic individual, lack of allergen-proof pillows or mattress encasements on the asthmatic individual’s bed, rodent activity, and cockroach activity. The cleaning index accounts for frequency of dusting and mopping/vacuuming the asthmatic individual’s room, washing the bed linens, washing the bed linens in hot water, and drying with hot air. Lower scores indicate the presence of fewer environmental triggers in the home.

### Analysis

For the current follow-up study, while in the home, field staff used iPads to collect and enter data into the RedCap system, a secure web-based application for building and managing databases. Our biostatistician reviewed the completed questionnaires and field observation forms and examined the data for consistency and outliers. The comparison of baseline to follow-up change in ACT and SQRQ score, healthcare utilization and environmental asthma risk indices was done using paired t-tests. The comparison of the percent of subjects experiencing respiratory symptoms was calculated by doing a two sample test of proportions on the difference of the percent’s. All statistical analysis was done using Statistical Analysis System (SAS) (Version 9.4) or Stata (Version 16) statistical software.

## Results

Our initial multifaceted environmental and educational intervention study with older adults (OAS) involved 86 lower income adults 62 years of age or older [20]. However, we collected data in this follow-up study (OAFS) on only 60 older adults. The remainder had either died, entered long-term care facilities, moved, or chose not to participate. As in our original multifaceted intervention study, this sub-cohort was primarily female, white, unmarried, and non-Hispanic. Most had less than a high school education and had a household income less than the area median. In addition, 19 participants also had a self-reported COPD diagnosis and two of those 19 currently smoke. Three additional participants currently smoke and two participants have someone living with them who smoke. On average the time between the end of the prior intervention study and the follow-up was 2.3 years (standard deviation 0.5 range 1.1–3.1 years) (Table 1).

**Table 1:** Demographics of older adult participants

Baseline characteristic	Older adults	
	*n*	%
Sex		
Female	44	73.3
Male	16	26.7
Race ^a^		
Black	1	1.7
White	39	65.0
Asian	16	26.7
Unknown/other	4	6.7
Ethnicity		
Non-Hispanic	35	58.3
Hispanic	25	41.7
Marital status		
Married	5	8.3
Education		
Any college	14	23.3
High school	5	8.3
< High school	41	68.4
Household Income ^b^		
0–50% area median income	44	72.9
≥ 50% area median income	4	6.8
Smoker in primary home ^c^	7	11.9
COPD	19 ^d^	31.7

*N* = 60. Participants were on average 73.0 years old

^a^ Older adults were able to indicate their ethnicity and race separately

^b^ Income level was often unreported. Since the study population lived in public or privately owned subsidized housing, it is likely that most unreported income was in the 0–50% area median income (AMI) category

^c^ 5 subjects were current smokers, 2 subjects lived with current smokers

^d^ 2 of 19 COPD subjects were current smokers (included in 7 above)

We investigated whether this OAFS subset (*n* = 60) had health outcome results that were consistent with the original OAS cohort (*n* = 86) from the previous study. In the original OAS study [20], we found statistically significant improvements in self-reported environmental asthma triggers and health outcomes in the number of doctor visits, uses of antibiotics for chest illness, ACT scores, SGRQ respiratory symptoms and quality of life indicators. This follow-up sub-cohort (OAFS) of *n* = 60 from the OAS study had the same significant outcomes (Table 2). Likewise, for the asthma symptoms that make up the SGRQ symptom score, significant improvements were seen for shortness of breath, cough and respiratory attacks 3 or more times per week, as well as self-reported fair to very good health for both the full (OAS) and sub-cohort (OAFS) of n = 60 (Table 3). The only difference seen between the full (OAS) and sub-cohort was a non-significant 10% decrease in those reporting taking daily asthma medication in the sub-cohort, compared to a significant 11.2% decrease in the original full cohort.

**Table 2:** Elders’ health and environmental outcomes

Outcomes (*N* = 60)	OAS baseline	OAS final	OAS baseline to final difference ^a^	OAFS follow-up	OAS baseline to OAFS follow-up difference ^b^	OAS final to OAS follow-up difference ^c^
Past 12 months						
Emergency room visits	0.64	0.41	0.24	0.30	0.34	0.12
Doctor visits	0.69	0.41	0.49*	0.63	0.02	−0.22
Hospitalizations	0.39	0.22	0.17	0.28	0.10	−0.05
Time on antibiotics for chest illnesses	0.81	0.39	0.39	0.65	0.15	−0.26
SGRQ scales (previous 3 months)						
Symptoms	52.4	40.4	12.8*	46.0	6.39	−5.51
Activity	75.3	72.0	3.87	77.0	−2.07	−4.80
Impact	52.5	38.8	14.5*	48.1	4.44	−9.71*
ACT scores						
Previous 4 weeks	13.6	16.1	−2.6*	14.7	−1.1	1.39
Environmental indices						
Allergen index score	4.02	2.4	1.90*	4.57	−0.27	−2.17*
Cleaning index score	4.55	3.92	0.63*	3.98	0.57*	−0.07

^*^ Significant difference at *p* ≤ 0.05

^a^ Change is calculated as a difference in paired values (OAS Baseline - OAS Final)

^b^ Change is calculated as a difference in paired values (OAS Baseline - OAFS Follow-up)

^c^ Change is calculated as a difference in paired values (OAS Final - OAFS Follow-up)

**Table 3:** Elders’ change in respiratory outcomes severity indicators

Outcomes (*N* = 60)	OAS baseline [% of elders]	OAS final [% of elders]	OAS baseline to final difference ^a^ [difference]	OAFS follow-up [% of elders]	OAS baseline to OAFS follow-up difference ^b^ [difference]	OAS final to OAS follow-up difference^c^ [difference]
Past 3 months						
Cough	70.0	50.8	19.2*	63.3	6.7	−12.5
Phlegm	46.7	33.9	12.8	36.7	10.0	−2.8
Shortness of breath	63.3	31.0	32.3*	59.3	4.0	−28.3*
Episodes of wheezing	33.9	22.4	11.5	22.4	11.5	0
3+ respiratory attacks	28.8	9.1	19.7*	18.3	10.5	−9.2
Currently						
Take daily asthma medications	96.4	86.4	10.0	71.7	24.8*	14.8*
Have rescue medications for asthma	85.2	74.6	10.6	91.4	−6.2	−16.8*
Health: fair to very good	63.3	79.7	−16.3*	72.9	−9.6	6.8

^*^ Significant difference at *p* ≤ 0.05

^a^ Change is calculated as a difference in paired values (OAS Baseline - OAS Final)

^b^ Change is calculated as a difference in paired values (OAS Baseline - OAFS Follow-up)

^c^ Change is calculated as a difference in paired values (OAS Final - OAFS Follow-up)

Our main question was whether the improvements from the baseline of the original OAS study had been maintained in the period after completion of the intervention study. We first examined the changes from baseline of the original OAS intervention study to the OAFS study. We found that there were no statistically significant differences between the OAS baseline and OAFS. However, all health care utilization metrics were lower than the OAS baseline at the OAFS follow-up (emergency room visits, doctor visits, hospitalizations, and times on antibiotics for chest illnesses) (Table 2). Likewise, although there were no significant differences, at the OAFS follow-up the SGRQ scores were better than at OAS baseline for symptoms and impact on quality of life, but not for activity level (Table 2). ACT scores were non-significantly better at OAFS follow-up than at OAS baseline. Although not significantly different, the environmental indices for indoor allergens worsened when comparing the original OAS baseline to the OAFS follow-up. However, the cleaning index was significantly better at OAFS follow-up compared to the baseline of the original OAS study (Table 2).

Within the SGRQ score for respiratory symptoms are a variety of symptom severity indicators which at the OAFS follow-up were not significantly different from the original OAS baseline scores, except for the percent taking daily asthma medications which showed a significant decrease of 25% (Table 3). Other severity indicators, though not significantly different, showed fewer subjects experiencing symptoms of cough, phlegm, shortness of breath, wheezing or respiratory attacks and more having rescue medications and reporting fair to very good health relative to the OAS baseline (Table 3).

These findings of generally better health at the OAFS than at the original OAS baseline are tempered by the findings that most indicators had declined from the final assessment of the OAS intervention study to the OAFS. Although not significantly poorer than at the original OAS final assessment, the number of doctor visits, hospitalizations and times on antibiotics had increased in the ensuing period after the intervention study up to the OAFS follow-up. The SGRQ scale for impact on quality of life was significantly worse at the OAFS relative to the final assessment of the OAS, while the SGRQ symptom and activity scales and the ACT score were also poorer, although non-significantly (Table 2). The SGRQ respiratory severity outcomes were worse from the final OAS final assessment to the OAFS follow-up for cough, phlegm, shortness of breath, respiratory attacks, and overall fair to very good health, however only shortness of breath reached statistical significance. At the OAFS follow-up statistically fewer subjects reported taking daily medications and more reported having rescue medications. The environmental indices at the OAFS follow-up were worse than at the OAS final assessment, though only the allergen index was significantly different.

## Discussion

Massachusetts has higher asthma rates than other regions of the United States [27]. In the Commonwealth of Massachusetts, the asthma rate among adults over 65 is reported to be 8.4%, and out of all age groups, hospital stays are the longest among seniors that have asthma (average of 4.7 days) [28] and the 2nd highest rate of hospitalization due to asthma [29]. The city of Lowell’s population was 106,519 in the 2010 U.S. Census, of which 47.2% were minority residents with 11.3% of the residents identified as of Puerto Rican descent (the largest subset of the 17.3% Hispanic residents) [30]. In our OAFS sub-cohort, 42% reported Hispanic as ethnicity and/or race. According to the National Health Statistics report on asthma prevalence in the United States, those of Puerto Rican descent have the highest asthma rate, twice the rate of the general population (16.6% versus 8.2%), as well as higher hospitalization and emergency room visits [27, 31].

In the original OAS study [20] (and this OAFS sub-cohort) we found a significant improvement from the baseline to the final assessment for a number of outcomes (reductions in self-reported environmental asthma triggers and health improvements in the number of doctor visits, uses of antibiotics for chest illnesses, ACT scores, SGRQ respiratory symptoms and quality of life indicators.) We also found significant improvements in the asthma symptoms that make up the SGRQ symptom score (shortness of breath, cough and respiratory attacks 3 or more times per week, as well as self-reported fair to very good health) for both the full OAS cohort and this sub-cohort.

The main finding of this study was that, with the exception of the SGRQ activity score, all subjects had better health outcomes at the OAFS than at the original OAS baseline. However, only the reduction in the percent of seniors taking daily asthma medications was statistically significant. Nevertheless, these findings suggest that the health impacts of the multifaceted environmental and educational intervention of the original OAS study were partially maintained by the subjects over the intervening period.

Although the outcomes at the OAFS did not decline to their OAS baseline level or below (except for SGRQ activity score), we did find a degradation in the outcomes from the improvements documented in the OAS final assessment to the OAFS follow-up. All outcomes, with the exception of emergency room visits and episodes of wheezing, were poorer at the follow-up than at the end of the OAS intervention study. Although none of these differences, except for SGRQ impact and shortness of breath episodes, were statistically significant, they nevertheless suggest that the multifaceted educational and environmental intervention could benefit from “booster” visits. These booster visits could include education on asthma management and medication and recognizing asthma triggers, provision of items to mitigate asthma triggers, such vacuums with HEPA filters, less-toxic cleaning alternatives, mattress and pillow encasements and trash cans with covers. Further study to evaluate this hypothesis is needed, as are estimates of the health care cost savings from periodic home interventions.

As in the OAS, this sub-cohort represents a generally sicker population and hence the multifaceted educational and environmental interventions may have shown a stronger benefit. All SGRQ scores were higher than general population norms for the age group 60–69 (population average symptom score 12.54, activity score 17.95, impact score 7.23) [32]. Also, we did see a significant decrease (worsening) in the SGRQ impact score from the OAS final assessment to the OAFS. This score accounts for quality of life and psychosocial stressors resulting from respiratory illnesses and medication use. It is possible that this change in score is due to the progression of their disease, however they did not regress back to the OAS baseline level. There was a significant decrease from the OAS final assessment to the OAFS in the percent of subjects taking daily asthma medications and an increase in those who had rescue medications for asthma. In the OAS 58% participants reported using steroidal medications daily in the past 12 months and 64% reported using non-steroidal medications daily in past 4 weeks. The reduction in those taking daily medications in the OAFS could reflect health improvements or changes in treatment protocols to more use of rescue medications with this population. However, it could also mean a lack of adherence to their treatment plan or the prohibitive cost of medication.

Since the allergen index score was worse at the OAFS than at the original OAS baseline or final assessments, it may be that some of the environmental interventions had fallen away. For example, 19 % of the participants no longer had the HEPA filtered vacuum provided during the original OAS intervention. Seven of the subjects who had no cockroaches at the original OAS final assessment had roaches at the OAFS follow-up, and 11 subjects who had no rodents at the original OAS final assessment had rodents at the OAFS follow-up. We found that some members of this cohort were often hesitant to inform housing managers about unit deficiencies or had language or communication limitations. Our Community Health Workers (CHWs) were often asked by participants to contact housing managers about deficiencies such as pest or mold problems.

During the original intervention study we found that some of the elder participants were unable to put the mattress encasements for dust mites onto the bed by themselves and/or unwilling to have mattress encasements put on the bed by the CHW during the home visits. Several elders preferred to have a family member help put the mattress covers on, and in some cases the mattress covers were not used at all. Nevertheless, by the final visit of the original OAS 81% of the elder participants were using mattress encasements, however by the OAFS follow up only 40% were using mattress encasements.

Limitations of this study include our inability to track and recruit all of the original OAS participants (*n* = 86) into the OAFS. The resulting number of subjects (*n* = 60) undoubtedly impacted our statistical power to quantify differences between the outcomes in the original OAS study and the OAFS, as well prohibiting evaluation of changes in medication usage due to the diversity of medication types among this subset. We did not have a control group in our original OAS study because our funder’s goal was to maximize intervention benefits and previous work in children suggested it would be unethical to limit the positive impacts likely to be seen among older study participants. Since the questionnaires on health outcomes covered the previous 3–12 months, recall bias was possible, especially with an older cohort. In addition, the questionnaire results were not validated against medical records for accuracy regarding healthcare utilization or confirmation of the asthma diagnosis by a general practitioner or pulmonologist. We also lacked objective measures, such as pulmonary function measurements to measure intervention impact. Although we collected data on self-reported COPD diagnosis and current smokers, we lacked data on who previously smoked or never smoked.

## Conclusion

Asthma continues to be a significant health problem for U.S. older adults especially in urban environments. Environmental asthma triggers include both indoor and outdoor sources. Allergens, dust, pets, mold, and tobacco smoke are known indoor asthma triggers. Our previous study demonstrated that a relatively low-cost comprehensive home educational and environmental intervention can significantly improve the health and emotional well-being of diverse, low-income older adults with asthma. In this OAFS we provide evidence suggesting that the health outcome improvements from these interventions, while remaining better than pre-intervention, had declined over time. However, there were few statistically significant differences from the original OAS baseline and final to the OAFS. We hypothesize that follow-up “booster” educational and environmental interventions are needed to sustain the health benefits achieved in the initial intervention. However, this will need further study with a larger population to verify; cost analyses should be done to quantify the health care spending benefits of periodic interventions. A larger follow-on study could also benefit from the use of objective measures of health outcomes including medical records to verify asthma status and medication use, health care utilization, and objective measures of health status such as pulmonary function tests.

## Data Availability

The datasets used and/or analyzed during the current study are available from the corresponding author on reasonable request.

## Abbreviations

ACT: Asthma Control Test
AMI: Area median income
ATA: Asthma trigger activities
BCHAP: Bureau of Community Health and Prevention
CDC: Center for Disease Control and Prevention
CHW: Community health workers
COPD: Chronic obstructive pulmonary disease
HEPA: High efficiency particulate air
HUD: United States Department of Housing and Urban Development
IRB: Institutional Review Board
OAFS: Older Adult Follow-up Study
OAS: Older Adults Study
SAS: Statistical Analysis System
SCHER: Scientific Committee on Health and Environmental Risks
SGRQ: Saint George’s Respiratory Questionnaire
WHO: World Health Organization
